# CRISPR/Cas9 for hepatitis B virus infection treatment

**DOI:** 10.1002/iid3.866

**Published:** 2023-05-10

**Authors:** Bo Cai, Shixue Chang, Yuhan Tian, Shuai Zhen

**Affiliations:** ^1^ Center of Medical Genetics Northwest Women's and Children's Hospital Xi'an Shaanxi PR. China; ^2^ Center for Translational Medicine The First Affiliated Hospital of Xi'an Jiaotong University Xi'an Shaanxi PR. China; ^3^ Genetic Disease Diagnosis Center of Shaanxi province Xi'an Shaanxi PR. China

**Keywords:** cccDNA, CRISPR/Cas9, HBV, nanoparticles

## Abstract

Hepatitis B virus (HBV) infection remains a global health challenge. Despite the availability of effective preventive vaccines, millions of people are at risk of cirrhosis and hepatocellular carcinoma. Current drug therapies inhibit viral replication, slow the progression of liver fibrosis and reduce infectivity, but they rarely remove the covalently sealed circular DNA (cccDNA) of the virus that causes HBV persistence. Alternative treatment strategies, including those based on CRISPR/cas9 knockout virus gene, can effectively inhibit HBV replication, so it has a good prospect. During chronic infection, some virus gene knockouts based on CRISPR/cas9 may even lead to cccDNA inactivation. This paper reviews the progress of different HBV CRISPR/cas9, vectors for delivering to the liver, and the current situation of preclinical and clinical research.

## Graphical abstract

This paper reviews the progress of different HBV CRISPR/cas9, vectors for delivering artificial potential therapeutic CRISPR sequences to the liver. This paper reviews the current situation of preclinical and clinical research.

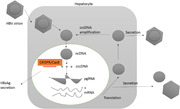

## 1 INTRODUCTION

The problems caused by chronic hepatitis B virus (HBV) infection continue to pose a major challenge to global health.^1^, ^2^, ^3^ Although an effective preventive vaccine is available, it is of little use to people chronically infected with HBV. In addition, the existing treatment methods have limited efficacy and can not reliably eliminate all replication intermediates of the virus.^4^ The persistence and difficulty in clearing of cccDNA (coupled closed loop DNA) and genomic integrated DNA. CCcDNA is almost the most important link in the life cycle of hepatitis B virus, and it is a template for virus replication and amplification. Once the patient's resistance declines, hepatitis B virus will make a comeback accordingly. In addition, hepatitis B virus DNA integrated into human hepatocyte genome may also start protein expression and viral nucleic acid replication in some cases.

cccDNA is a stable replication intermediate that serves as a template for pregenomic RNA (pgRNA) and viral protein‐encoded mrnas.^5^, ^6^ Naturally, extra chromosomal DNA (such as cccDNA) is a target for transcriptional silencing through the maintenance of the cell structure of chromosome complex.^7^, ^8^ Another compact arrangement is to embed regulatory elements such as promoters and enhancers in the virus ORF.^9^, ^10^

## 2 CRISPR/CAS9 AND HBV TREATMENT

Treatment for HBV infection has been identified. Functional therapy describes the state of the patient after HBsAg elimination, with or without seroconversion, but is usually associated with the persistence of cccDNA^1^, ^11^ Functional therapy is currently considered to be the realistic goal of treatment strategies. However, complete elimination of the virus may require combination therapy, which is the ultimate goal of HBV treatment.^12^ One difficulty in assessing treatment success is the lack of appropriate biomarkers to measure cccDNA in the liver. Some of the potentially useful studies recently described include assessments of core associated HBV antigens (HBcrag) and serum viral RNA. These show promise, but need to be thoroughly verified.^13^, ^14^

## 3 GENE EDITING STRATEGY

**Figure 1:**
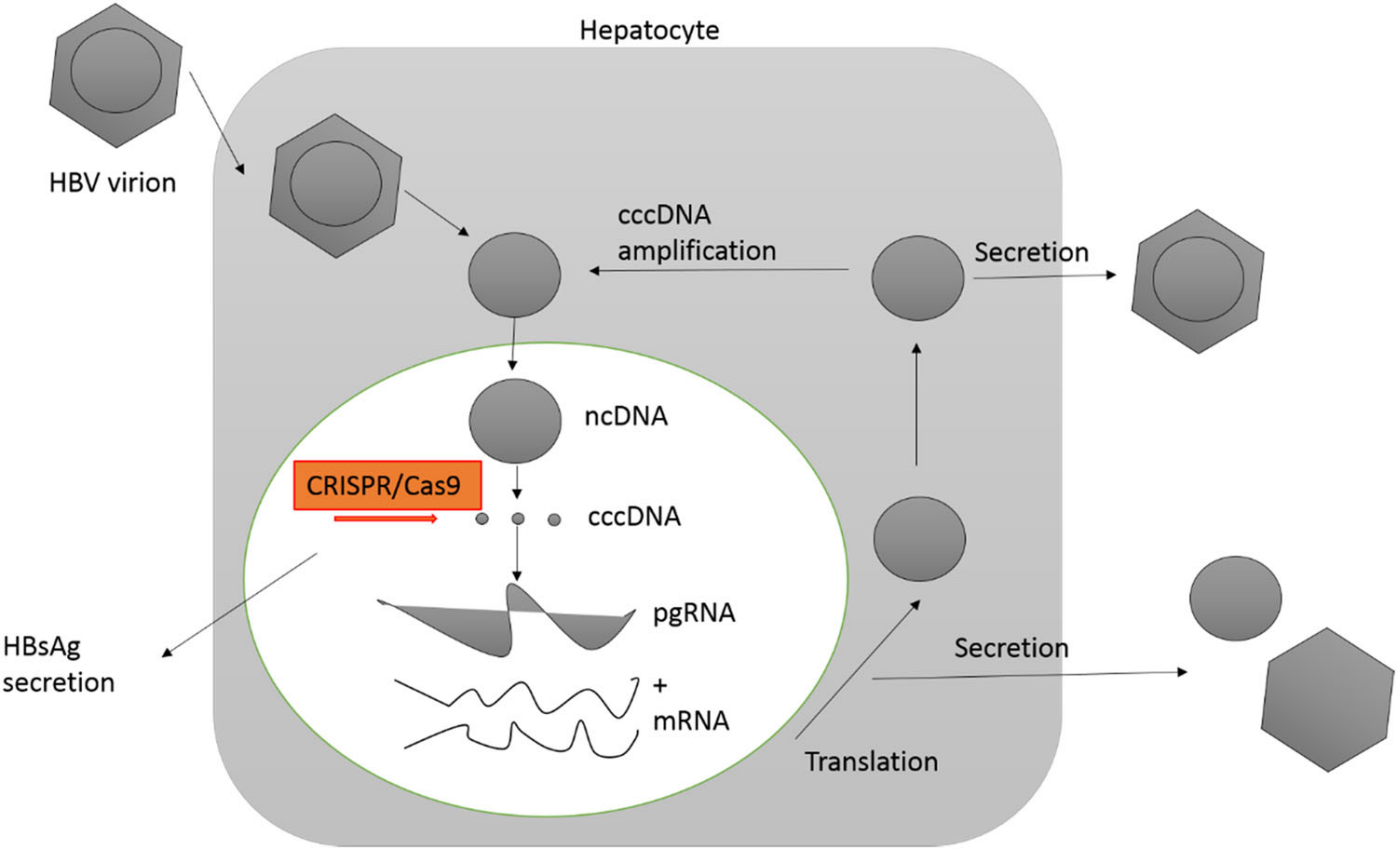
Application of CRISPR/Cas9 in targeted HBV genome editing. HBV, hepatitis B virus.

Recently, the development of new sequence‐specific nuclease techniques has led to the development of several novel anti‐HBV therapeutic strategies. These include zinc finger nucleases, talens (transcription‐activator‐like effect nucleases) and CRISPR/cas9 systems, which have had some success in reducing viral DNA and cccDNA levels in cell cultures and animal models through viral gene editing. Most antiviral gene‐editing therapies, as mutagenic agents, cause DNA damage at sequence‐specific sites of cccDNA when cells enter. DNA repair via nonhomologous recombination of these sites is error‐prone, leading to insertion/deletion into cccDNA (Figure 1).

CRISPR/cas9 was first evaluated in the treatment of HBV in 2014.^15^ Several studies have demonstrated successful inhibition of viral replication and production of viral markers, such as HBsAg, by simultaneous application of multiple small guide Rnas targeting different regions of the HBV genome. Our previous study showed that the effects of the CRISPR/CRISPR‐associated Cas9 system that was targeted to the surface antigen (HBsAg)‐encoding region of HBV, both in a cell culture system and in vivo. The HBsAg levels in the media of the cells and in the sera of mice analyzed by a quantitative enzyme‐linked immunosorbent assay. The HBV DNA levels assessed by quantitative PCR and HBsAg expression in mouse livers assessed by an immunohistochemical assay. The amount of HBsAg secreted in the cell culture and mouse serum reduced by CRISPR/Cas9 treatment. Immunohistochemistry analyses showed almost no HBsAg‐positive cells in the liver tissue of CRISPR/Cas9‐S1+X3‐treated mice. The CRISPR/Cas9 system efficiently produced mutations in HBV DNA. Thus, CRISPR/Cas9 inhibits HBV replication and expression in vitro and in vivo and may constitute a new therapeutic strategy for HBV infection. In general, the preclinical study of gene editing technology is promising. However, some challenges remain. First, drug delivery methods that can maximize efficacy and reduce immune response to therapeutic gene editing still being developed.^16^ After treatment with CRISPR/cas9 system, virus escape mutants have also attracted attention. Finally, the adverse events of these drugs need to be carefully studied, including nonspecific cleavage of host chromosomes and chromosome instability caused by cleavage of viral DNA integrants.

## 4 NONVIRAL VECTORS/VIRAL VECTORS FOR DELIVERY OF CRISPR/CAS9

An important goal of CRISPR/cas9 based HBV therapy is targeted delivery to the liver.^17^ Several delivery strategies have been studied, including synthetic nonviral preparations and recombinant viral vectors for CRISPR/cas9 delivery.^18^, ^19^

## 5 NONVIRAL VECTORS

Lipid nanoparticles (LNPS) were used to deliver CRISPR/cas9 in many earlier studies. These vectors include nucleic acid binding lipids and other compounds assembled into LNPS.^20^ A uniformly small LNP (<100 nm diameter) is important to avoid isolation during the cycle and promote access to hepatocytes through the sinusoidal window. The characteristic of promoting effective endosomal escape also needs to increase the delivery of cytoplasmic CRISPR/cas9.^21^, ^22^

In earlier studies, chemically modified HBV targeted gRNAs integrated into stable nucleic acid lipid particles (SNALPs) and injected intravenously into HBV replicating mice. Compared with the unprepared CRISPR/cas9, the half‐life of CRISPR/cas9 prepared by this SNALPs in the liver is longer and the curative effect is better.^15^ A decrease in serum HBV DNA detected within 6 weeks after weekly administration. Pegylated nanoparticles successfully used to target unmodified CRISPR/Cas9 to the liver. After repeated systemic administration, a threefold reduction in HBV replication markers observed in HBV transgenic mice within 28 days. The guanidine‐modified GrNA‐targeted Xphilic lipid plexus can also effectively block HBV replication.^23^, ^24^

Recently, conjugated CRISPR/cas9 has gained momentum in preclinical and clinical applications. CRISPR/cas9 in combination with N‐acetylgalactose amino‐amino (GalNAc/NAG) has emerged as a popular choice for liver targeted drug delivery. The binding of CRISPR/cas9 to ligands derived from liver cells enables the uptake of the desialoglycoprotein receptor (ASGPR) by liver cells, enabling liver‐specific delivery of CRISPR/cas9 in vitro and in vivo.^25^ A single combined injection of GalNAc combined with melittin like peptide (NAG‐MLP) and cholesterol combined with HBV targeted gRNA successfully reduced the expression of HBV RNA, DNA and protein in mice. Two similar bottles of preparations used in the famous candidate clinical trial arc‐520 (arrow drug), in which two different cholesterol binding CRISPR/cas9 were mixed with NAG‐MLP before injection. Arc‐520 initially showed promising preclinical and clinical results, but in relevant safety studies in nonhuman primates, the lethal toxicity of the nag‐mlp version of EX1 dynamic poly conjugate (DPC) vehicle led to the suspension of arc‐520 clinical trial. In a recent development, GalNAc modified to produce a tripeptide GalNAc ligand, which can achieve robust and lasting gene silencing in the liver after subcutaneous administration in mice. In the process of further improving the formula, CRISPR/cas9 modification significantly enhanced the stability of triple stranded GalNAc CRISPR/cas9conjugate in mice and nonhuman primates. This effect may be the result of preventing the degradation of extracellular enzymes. Strategic localization of chemical modifications, such as 2ʹ‐deoxy‐2ʹ‐fluorine and 2ʹ‐o‐methylribose, also improved CRISPR/cas9 potency and knocking‐out time in nonhuman primates. Interestingly, fully modified CRISPR/cas9 seems to be more suitable for conjugate mediated transmission in vivo, regardless of CRISPR/cas9 sequence or conjugate type.^24^, ^26^

## 6 VIRAL VECTORS

The virus has evolved an efficient cell transduction mechanism, which has been used to produce virus vectors.^27^ Usually, these vectors have replication defects and lack the viral components necessary for replication after infected cells. Transgene is coupled to viral vector packaging signal, and vector particles are generated in packaging cells by expressing essential components in trans viral vector.^15^ Recombinant viral vectors usually interact with cell surface molecules to promote endocytosis, and then affect a series of events, eventually leading to transgene transmission and expression.

Recombinant lentivirus vectors (LVS), adenovirus vectors and adeno‐associated virus vectors (AAV) all show good liver transduction efficiency, so they are a reasonable choice for delivering anti HBV sequences.^28^, ^29^ However, not all vectors are suitable for carrying HBV targeted silencing expression boxes. Adenovirus vectors usually exhibit a high degree of innate immune stimulation, resulting in relatively short‐term transgene expression. Lentiviral vectors transduce adult hepatocytes with low efficiency in vivo and carry cancer risk due to chromosome integration.^30^

AAV has become a good candidate for gene therapy. Subsequent studies alleviated initial concerns about the carcinogenic potential of AAV.^31^, ^32^ Some preclinical studies on HBV and other diseases have proved the safety and effectiveness of AAV^33^ in vivo. In addition, a recently published 1‐year study showed that glybera, an AAV based gene therapy drug for lipoprotein lipase deficiency, treated a patient, which confirmed the safety and effectiveness of AAV vector in human application. AAV has an uncapsulated icosahedral structure.^34^, ^35^ The characteristics of capsid proteins from different serotypes determine the characteristics of the carrier.^27^, ^36^, ^37^ Although scAAVs has a small transgenic ability, an important advantage of these vectors is that their transgenic expression speed is faster. In addition, the interesting observation that HBV infection enhances hepatocyte AAV transduction makes these vectors very suitable for delivering anti HBV CRISPR/Cas9 expression boxes.^38^

The use of AAV to provide anti HBV CRISPR/Cas9 has been widely explored. Delivery of HBV CRISPR/Cas9 using scAAV inhibited HBV replication in transgenic mice, which lasted for 10 months.^39^, ^40^ The use of AAV to deliver CRISPR/Cas9, as well as components of RNAi mechanisms, such as Argonaute 2 or sensory chain inhibitory RNA bait, can avoid saturation of endogenous RNAi pathway, reduce toxicity, improve specificity, and enhance efficacy.^41^

Despite its impressive efficacy and safety profile, rAAV is not without its drawbacks. The high prevalence of pre‐existing immunity to vectors derived from prevalent serotypes such as AAV2 or AAV8 leads to transient expression of transgenes in humans.^42^, ^43^ AAV capsid recombination, capsid de novo design, directed evolution, and silicon carrier synthesis are addressing this obstacle. An example of directed evolution is the use of mutant capsids produced by DNA rearrangement, exposed to multiple rounds of selection pressures by neutralizing antibodies. This has led to the selection of AAV vectors that evade immunity and transduction effectively in the liver. Another way to overcome the pre‐existing immunity of AAV capsids is to give transgenic capsids and decoy (empty) capsids.^44^, ^45^

Electronic construction of synthetic AAV vectors is a particularly promising method for avoiding pre‐existing immunity and has used to generate ancestral genes that code for synthetic AAV capsid variants, a method that requires several steps.^46^ The existing AAV capsids from different serotypes compared, and then the ancestral protein sequences determined by phylogenetic method. The predicted ancestral DNA sequence cloned and expressed in transfected mammalian cells and selected according to the capsid coding sequence that can package the AAV genome. Some encouraging results based on AAV capsid modification to improve liver transduction predict the progress of AAV in transmitting HBV Gene Silencers.^47^

## 7 FUTURE DIRECTIONS AND CONCLUSION

CRISPR/cas9 has shown to block HBV replication in vitro and in vivo. The persistence of knockout effect by expressing HBV targeted sequence is a very useful feature in the treatment of chronic diseases.^48^, ^49^ However, before this method is transformed into clinical application, it is necessary to successfully promote the technology to realize the cheap production of virus vector and avoid the effective transduction of host immunity and hepatocytes.^50^, ^51^ CRISPR/cas9 in nonviral agents is currently at a higher stage than HBV‐targeted viral vectors and is undergoing clinical trials. The available clinical data are promising and indicate that anti‐HBV CRISPR/cas9 is safe and well tolerated. Given the large number of chronic hepatitis B virus infections, an important potential advantage of these synthetic drug candidates is that they are easier to scale up and produce economically.^52^, ^53^, ^54^, ^55^

The progress in optimizing gene silencing effectors and identifying good HBV targets is impressive. Knockouts have been targeted to sequences that contain almost the entire HBV genome, but targeting specific viral sequences does not seem to produce particularly good results. Nevertheless, some viral targets may be more favored for reasons that are not fully understood.^56^, ^57^, ^58^ Further understanding of the cellular mechanisms of HBV infection and replication, especially those related to the biogenesis, transcription and degradation of cccDNA, may provide necessary insights for improving the selection of viral homologues. HBx has recently been shown to promote the degradation of chromosome (SMC) 5/6 complex structure maintenance. This effect is achieved by the action of cellular ubiquitin ligase and leads to enhanced transcription of cccDNA.^59^, ^60^ It will be interesting to determine whether CRISPR/Cas9 activators targeting HBx have a special inhibitory effect on transcription from cccDNA. Inhibiting cccDNA transcription, especially if sustained, will be a major achievement and achieve the functional treatment of HBV infection to a certain extent. Another advantage of targeting CRISPR/cas9 to HBx is that the sequence is common to all viral transcripts and can inhibit the expression of all viral proteins by targeting the gene.^61^, ^62^, ^63^

Developing new methods to prepare vectors, avoid host immunity, describe pharmacokinetics, eliminate unexpected target knockouts, and determine biomarkers of cccDNA in vectors are current priorities.^64^ In addition, existing animal models of HBV infection have limitations, and testing drug candidates in Settings close to natural chronic HBV infection will facilitate drug development. Achieving these goals will provide momentum. However, the overall progress in the field suggests that effective silencing of viral transcripts produced by cccDNA in chronic HBV infection is achievable and has therapeutic potential. Results from current and future clinical trials are eagerly awaited. The results of these studies will pave the way for achieving functional therapeutic goals for chronic HBV infection.^65^, ^66^, ^67^

## 8 CONCLUSION

For future applications of CRISPR/Cas9 technology to eradicate HBV virus libraries, safety, efficacy and specificity will be the focus. Research efforts using CRISPR/Cas9 will now focus on primate models and phase I clinical trials. From the point of view of this review, CRISPR/Cas9 technology holds great promise for HBV eradication. The CRISPR/Cas9 system could be used in the clinical treatment and prevention of HBV, as the FDA has approved the use of the technology in multiple clinical trials.

## AUTHOR CONTRIBUTIONS

Shuai Zhen conceptualized the study. Shuai Zhen wrote the manuscript. All authors read and approved the fnal manuscript.

## CONFLICT OF INTEREST STATEMENT

The authors declare no conflict of interest.
